# Crossover-effects in technical skills between laparoscopy and robot-assisted surgery

**DOI:** 10.1007/s00464-023-10045-6

**Published:** 2023-04-25

**Authors:** Sem F. Hardon, E. Willuth, A. Masie Rahimi, F. Lang, Caelan M. Haney, Eleni A. Felinska, Karl-Friedrich Kowalewski, Beat P. Müller-Stich, Donald L. van der Peet, Freek Daams, F. Nickel, Tim Horeman

**Affiliations:** 1 Department of Surgery, Amsterdam UMC – VU University Medical Center, ZH 7F 005 De Boelelaan 1117, 1081 HV Amsterdam, The Netherlands; 2 Department of Biomechanical Engineering, Delft University of Technology, Delft, The Netherlands; 3 Department of General, Visceral and Transplantation Surgery, Heidelberg University, Heidelberg, Germany; 4 Amsterdam Skills Centre for Health Sciences, Amsterdam, The Netherlands

**Keywords:** Robot-assisted surgery, Laparoscopy, Technical skills, Assessment, Crossover effects, Patient safety

## Abstract

**Introduction:**

Robot-assisted surgery is often performed by experienced laparoscopic surgeons. However, this technique requires a different set of technical skills and surgeons are expected to alternate between these approaches. The aim of this study is to investigate the crossover effects when switching between laparoscopic and robot-assisted surgery.

**Methods:**

An international multicentre crossover study was conducted. Trainees with distinctly different levels of experience were divided into three groups (novice, intermediate, expert). Each trainee performed six trials of a standardized suturing task using a laparoscopic box trainer and six trials using the da Vinci surgical robot. Both systems were equipped with the ForceSense system, measuring five force-based parameters for objective assessment of tissue handling skills. Statistical comparison was done between the sixth and seventh trial to identify transition effects. Unexpected changes in parameter outcomes after the seventh trial were further investigated.

**Results:**

A total of 720 trials, performed by 60 participants, were analysed. The expert group increased their tissue handling forces with 46% (maximum impulse 11.5 N/s to 16.8 N/s, *p* = 0.05), when switching from robot-assisted surgery to laparoscopy. When switching from laparoscopy to robot-assisted surgery, intermediates and experts significantly decreased in motion efficiency (time (sec), resp. 68 vs. 100, *p* = 0.05, and 44 vs. 84, *p* = 0.05). Further investigation between the seventh and ninth trial showed that the intermediate group increased their force exertion with 78% (5.1 N vs. 9.1 N, *p* = 0.04), when switching to robot-assisted surgery.

**Conclusion:**

The crossover effects in technical skills between laparoscopic and robot-assisted surgery are highly depended on the prior experience with laparoscopic surgery. Where experts can alternate between approaches without impairment of technical skills, novices and intermediates should be aware of decay in efficiency of movement and tissue handling skills that could impact patient safety. Therefore, additional simulation training is advised to prevent from undesired events.

**Supplementary Information:**

The online version contains supplementary material available at 10.1007/s00464-023-10045-6.

Robot-assisted surgery (RAS) is increasingly being performed [1–3]. Consequently, surgeons are expected to master a different spectrum of technical skills. While laparoscopy surgery (LS) is still frequently being performed, it occurs that surgeons switch between approaches in the operating room (OR) with short periods of time in between. Utilizing a robotic system does impact the posture of the surgeon leading to different muscle stress locations and differences in fatigue progression [4, 5]. Moreover, RAS gives a different touch and feel of the instruments, the tissues and its environment due to the absence of tactile and haptic feedback [6–8].

Implementation of RAS depends on hospital resources and the opportunity to train in a simulated environment, without compromising patient safety [9–14]. Whether technical skills are mastered before entering the OR depends on training time and individual assessment and feedback during training [15, 16]. Currently, research seems to focus on identifying the ideal learning curve its plateau, and, moreover, on how to assess whether this stage is entered for different kind of technical skills [17, 18]. Previous studies showed that laparoscopic skills, such as efficient use of instruments and safe tissue handling, can be objectively assessed in simulation training using performance parameters [19–22]. However, little is known about the transferability of these tissue handling skills when surgeons switch between LS and RAS [23, 24].

The aim of this study is to investigate the crossover effects when switching between approaches. It was hypothesized that intermediates, in the middle of the learning curve and subject to LS simulation training, tend to focus on efficiency of movement (i.e., speed and time), rather than tissue handling parameters (i.e., force exertion), resulting in high force-parameter outcomes [22, 25–28]. Also, based on the intuitiveness attributed to RAS, it was hypothesized that switching from RAS to LS will result in increased tissue handling forces, especially in the less experienced groups.

## Methods

### Study design

This international, multicentre, prospective, crossover study was conducted at the Heidelberg University Hospital (Germany, Heidelberg), the Amsterdam University Medical Centers (The Netherlands, Amsterdam) and the Amsterdam Skills Centre for Health Sciences (Amsterdam, The Netherlands).

### Participants

Participants were classified and divided into three groups based on their prior experience: *novices* consisting of junior residents (< 10 laparoscopic procedures, 0 robotic-assisted surgery procedures and no exposure to the system), *intermediates* consisting of senior residents and young surgeons (< 200 laparoscopic procedures, 0 robotic-assisted surgery procedures, 25 robotic knots) and *experts* consisting of attendees/robot experts (> 200 laparoscopic procedures, > 15 robotic-assisted surgery procedures as console surgeon, and > 50 robotic knots).

### Systems and hardware

Both the da Vinci Xi Surgical System (Intuitive Surgical Inc., Sunnyvale, California USA) at the OR and a the Szabo-Berci-Sackier laparoscopic box trainer (Karl Storz, Tuttlingen, Germany) were equipped with the validated ForceSense measurement system (MediShield B.V., Delft, the Netherlands) [19, 22, 29, 30]. This system recorded raw time, 3D motion, and 3D force data from the two Trendo trocar sensors and the ForceTRAP sensor, respectively, mounted in the lid of the box trainer and bottom plate. This system recorded performance parameters that have been proven to represent efficient instrument use and tissue handling skills [19, 22]. Implementing this system facilitated objective assessment of tool-tissue interaction and instrument handling skills [29, 30]. After implementation, this system recorded the following parameters each trial: total time required to finish the task, a set of maximum and mean interaction forces and the force volume during tissue manipulation (Table 1). After each trial, the data and a video log of the performed task were stored in an online database (ForceSense.NET, MediShield, Delft, The Netherlands). The participants used two needle drivers (BBraun Aesculap, Melsungen, Germany) with a Novosyn HR26 needle and a 70 cm 3/0 thread for needle driving, suturing and knot tying. For each trial a separate surgical suture was used, equating to twelve surgical sutures for each participant.

**Table 1:** Description of the objective performance metrics of the ForceSense

Parameter	Description
Task time	Total time needed to complete the task presented in seconds (s)
Maximum absolute force	The highest absolute force applied on the training task during tissue manipulation (N)
Mean non zero force Max impulse	The average force exerted on the training task during tissue manipulation (N) [19, 22, 29] The highest absolute force-over-time integral applied on the training task (N/s) [19, 22, 29]
Force volume	When viewing the forces in a 3d plane an ellipsoid is imagined. The force volume consists of the multiplication of the forces (and standard deviation) in the height, length and width of the ellipsoid [19, 22, 29]

### Protocol

**Fig. 1:**
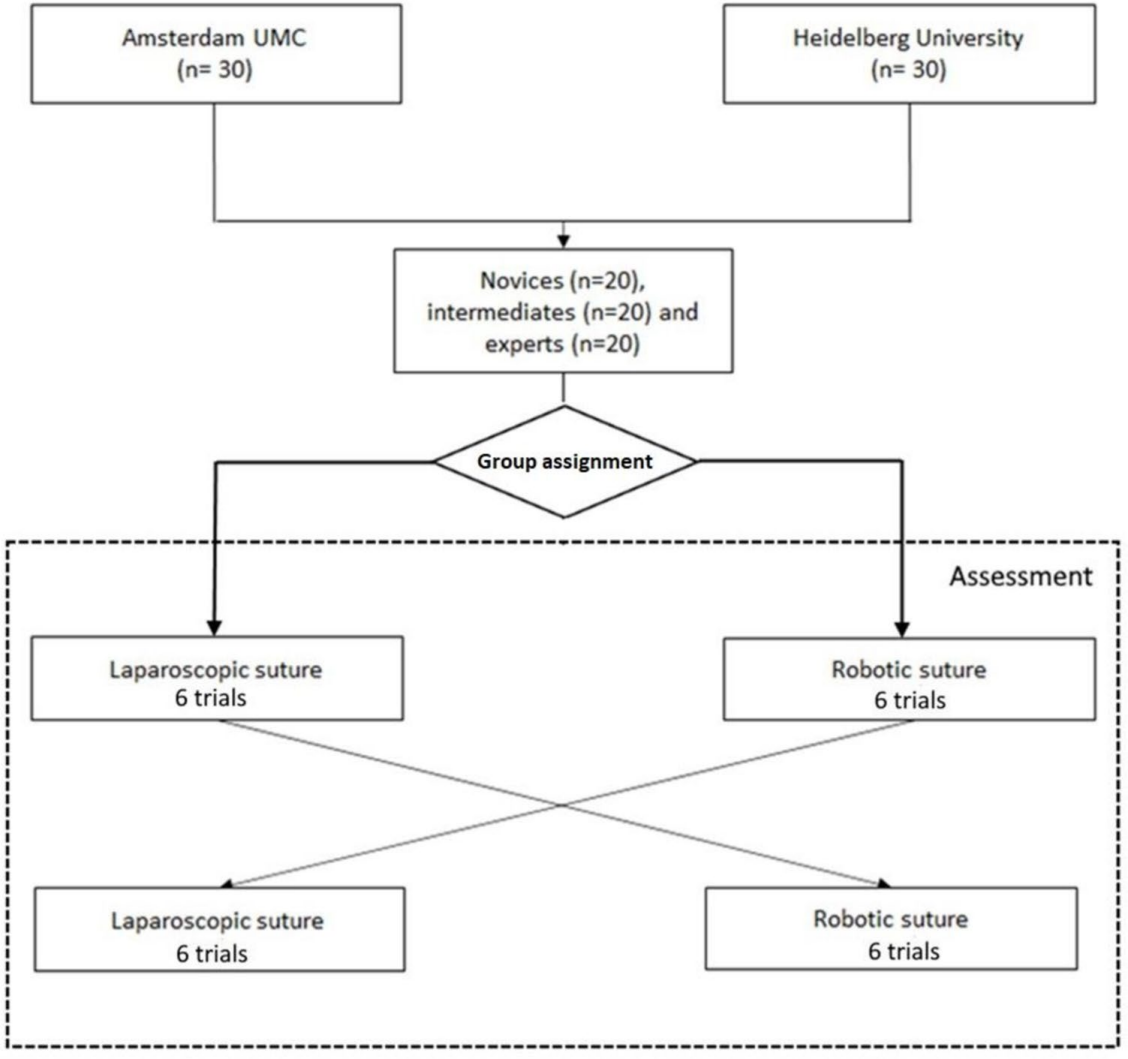
Crossover study design flow chart

The participants were assigned in two groups, one group which started with the robotic trials (RL group novices, intermediates and experts) and one group which started with the laparoscopic trials (LR group novices, intermediates and experts). Group assignment was not fully randomized as it was influenced by the availability of the robotic and laparoscopic training systems and logistics. All trainees received a brief verbal instruction on the da Vinci Surgical System. After group assignment, each trainee performed twelve trials of a standardized minimally invasive suturing task (three-throw square knot). For the LR group, the first six trials were performed using the laparoscopic box trainer, followed by six trials using the da Vinci Surgical System (Intuitive Surgical Inc., Sunnyvale, California USA). Vice versa for the RL group (Fig. 1). Each trial of the task was measured and stored in the online database separately. The local ethics committee at Heidelberg University approved the study protocol before inclusion of the trainees.

### Statistical analyses

Data were analysed using IBM SPSS statistics 26 (SPSS Inc., Chicago, Illinois USA). Descriptive statistics and frequency measurements were performed to determine the means, standard deviation and normal distribution of the data. The Shapiro–Wilk test was performed and the data were not normally distributed. GraphPad (Prism 9.0.0, San Diego, California USA) was used to visualize boxplots. Statistical comparison was done between the sixth and seventh trial to identify transition effects when subjects move from laparoscopy to robotic surgery or vice versa. Post hoc power analysis (power (1−*β*) of 0.80, two sided and a test significance level (*α*) of 0.05) was performed. An outcome with a *p* ≤ 0.05 was considered as statistically significant.

## Results

A total of 720 trials, performed by 60 participants, were included for analyses (20 novices, 20 intermediates, 20 experts). One participant in the novice group was left dominant. There were 19 females included (6 novices, 10 intermediates, 3 expert).

### Training effect

In the RL group (*N* = 33), novices reduced their maximum force (7.4 N vs 4.5 N, *p* = 0.01), maximum impulse (25.6 N/s vs 14.4 N/s, *p* = 0.01) and force volume (2.0 N vs 1.5 N, *p* = 0.01) between the first and the sixth RAS trials. The experts reduced their total completion time (110 s vs 76 s, *p* = 0.01) and maximum impulse (16 N/s vs 11 N/s, *p* = 0.05) in the RAS trials, and significantly improved all parameters in the LS trials (no. seven to twelve). A decrease in maximum forces (4.4 N to 3.1 N, *p* = 0.04), and maximum impulse from (16.8 to 9.8 N, *p* = 0.02) was observed. The intermediates had a reduced total completion time (91 s vs 80 s, *p* = 0.02) after the LS trials. In the LR group (*N* = 27), the novices (205 s vs 134 s, *p* = 0.01) and experts (82 s vs 44 s, *p* = 0.03) improved their total completion time. A detailed overview is provided in the supplemental files.

### Crossover effect

**Fig. 2:**
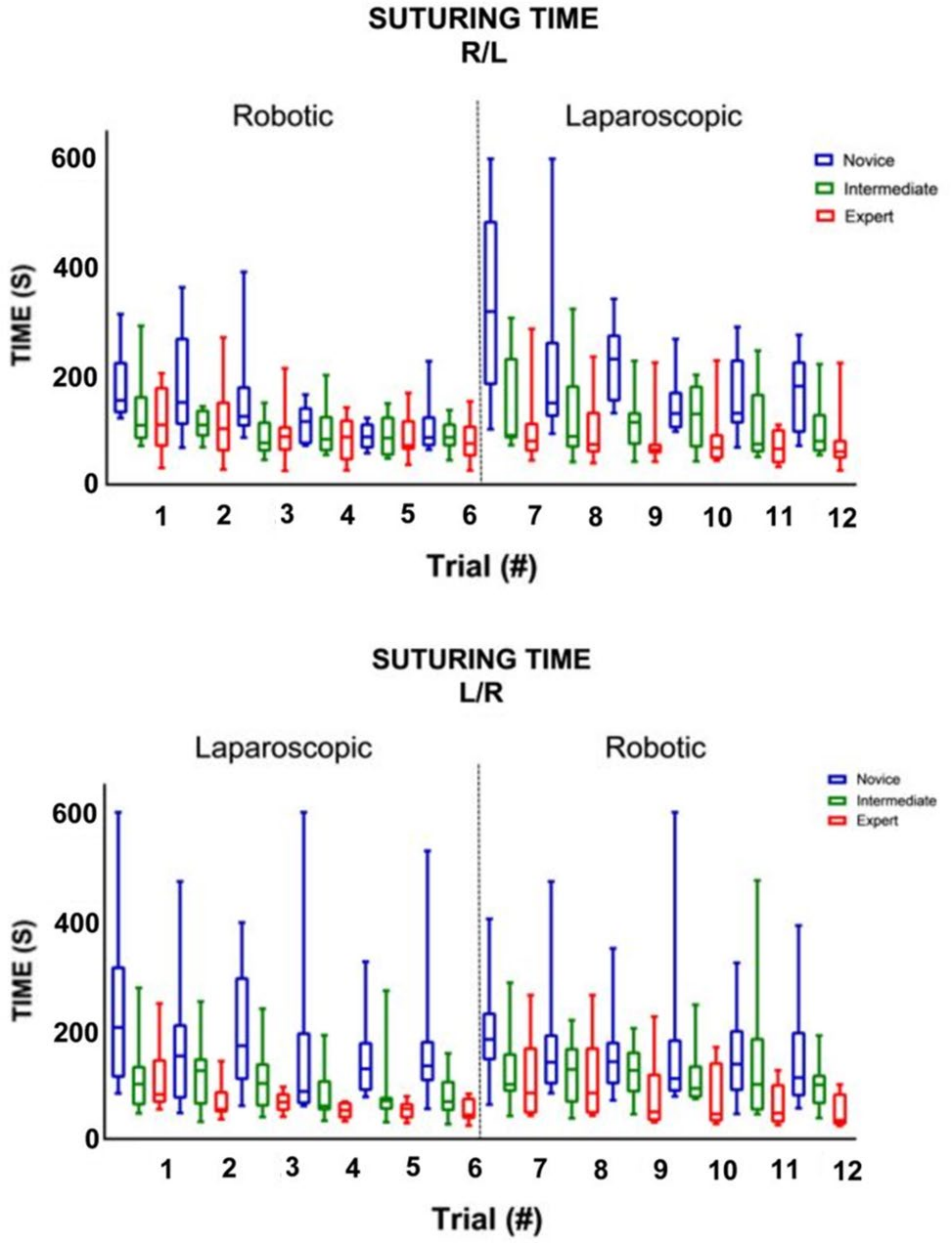
Laparoscopic and robotic suturing time (in seconds)

**Fig. 3:**
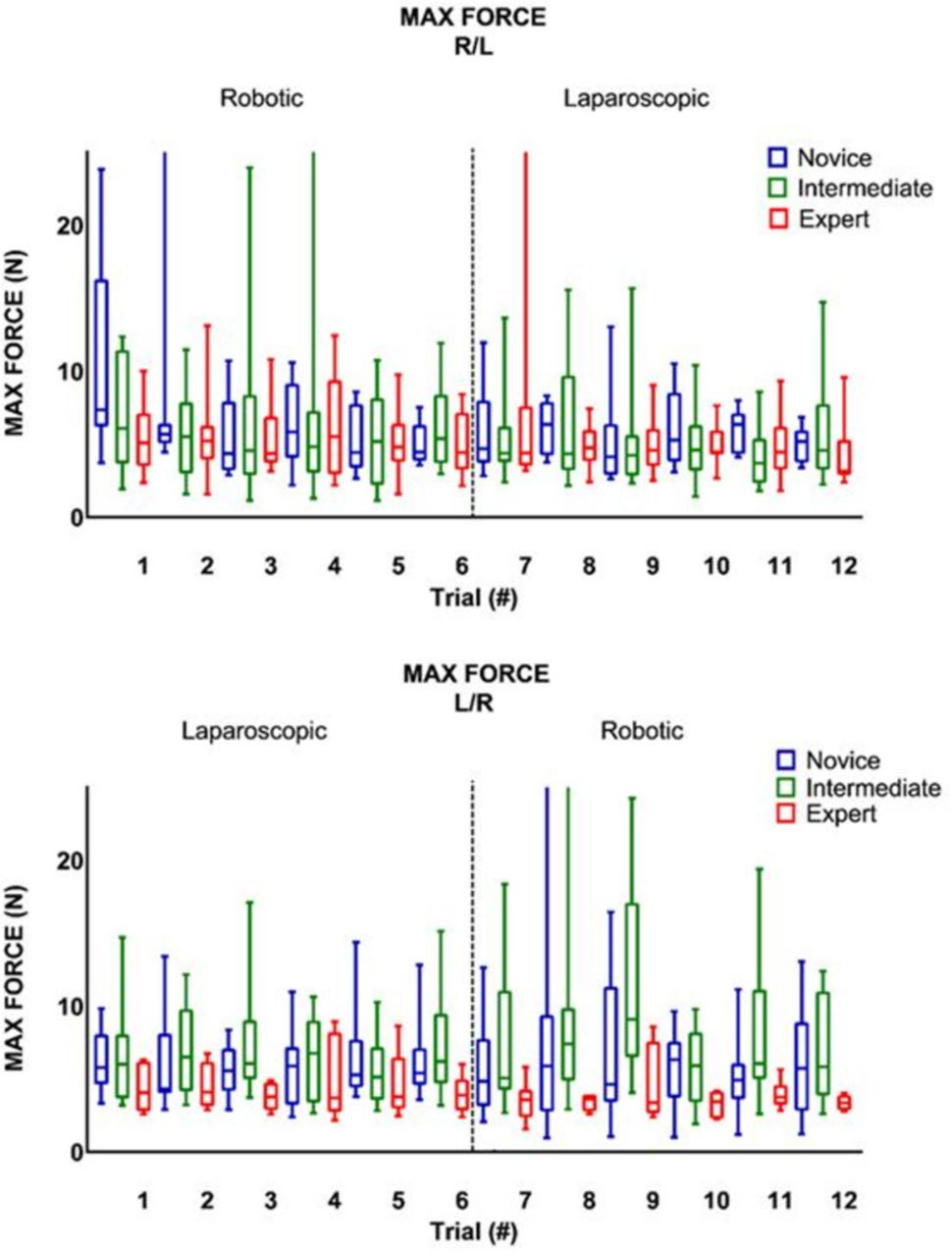
Laparoscopic and robotic suturing maximum absolute force (in N)

**Fig. 4:**
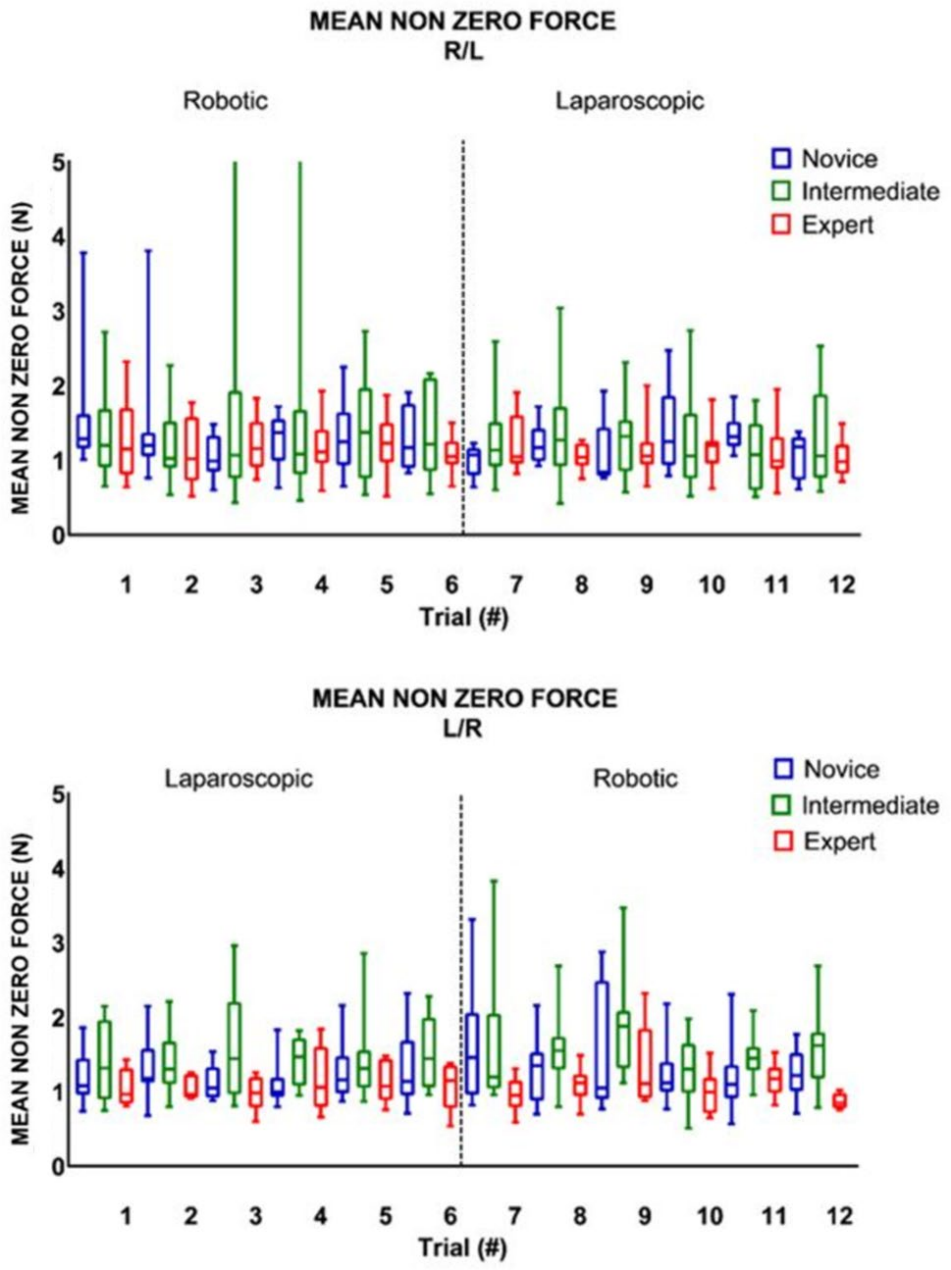
Laparoscopic and robotic suturing mean non zero force (in N)

**Fig. 5:**
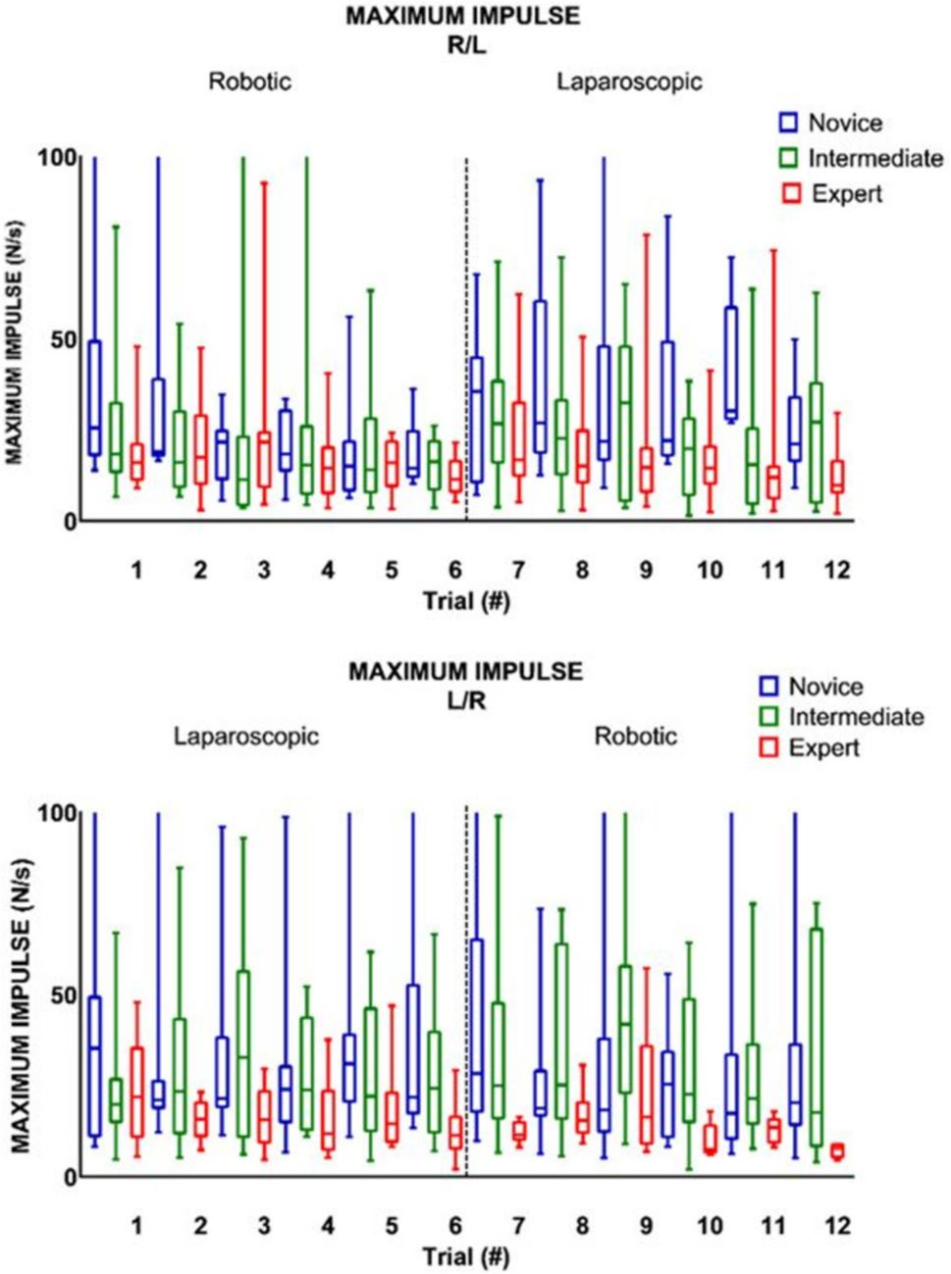
Laparoscopic and robotic suturing maximum impulse (in N/s)

**Fig. 6:**
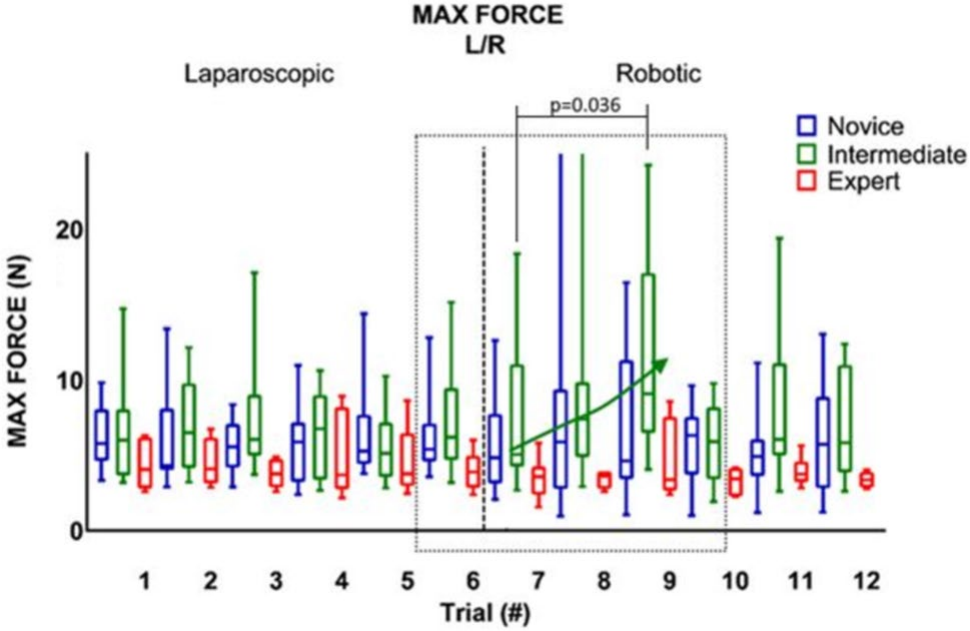
Strong effects maximum exerted forces occur for the participants with less experience after switching systems from LS to RAS

Table 2 shows, that when switching from RAS to LS (trial six and seven), there was no significant change in any of the parameter outcomes, except for maximum impulse (Figs. 2, 3, 4). The expert group increased their maximum impulse with 46% (11.5 N/s to 16.8 N/s, *p* = 0.05) (Fig. 5). When switching from LS to RAS, the total time was increased for intermediates (68.7 s vs. 100.2 s, *p* = 0.05) and for experts (44.2 s vs. 84.5 s, *p* = 0.05). However, an significant decrease in MaxForce of 8.5% was observed in the expert group (3.9 N vs. 3.6 N, *p* = 0.01). The comparison of the seventh and ninth laparoscopic and robotic suturing trials, provided in Table 3, shows that the max force levels of both the intermediates and novice group start to increase after the seventh trial (Fig. 6). A 78% increase inforce exertion is was observed between the 7th and 9th trial for the intermediates (5.1 N vs. 9.1 N, *p* = 0.04).

**Table 2:** Comparing the sixth and seventh laparoscopic and robotic suturing trials

	RAS—LS				LS—RAS			
	Trial 6	Trial 7	*Z* value	*p* value	Trial 6	Trial 7	*Z* value	*p* value
Time (s)								
Novice	86.72	274.10	− 1.82	NS	134.28	182.92	− 1.33	NS
Intermediate	87.31	90.82	− 1.68	NS	68.68	100.21	− 1.99	0.05
Expert	75.59	80.15	− 0.71	NS	44.17	84.52	− 1.99	0.05
Max. force (N)								
Novice	4.46	4.09	− 0.98	NS	5.40	4.84	− 1.07	NS
Intermediate	5.36	4.39	− 0.46	NS	6.21	5.07	− 0.05	NS
Expert	4.42	4.39	− 0.24	NS	3.90	3.57	− 2.67	0.01
Mean NZ force (N)								
Novice	1.17	1.07	− 1.54	NS	1.14	1.46	− 1.69	NS
Intermediate	1.22	1.14	− 0.05	NS	1.45	1.20	− 0.05	NS
Expert	1.05	1.05	− 0.77	NS	1.15	0.95	− 1.10	NS
Max. impulse (N/s)								
Novice	14.43	35.63	− 1.1	NS	21.85	28.38	− 0.98	NS
Intermediate	16.32	26.75	− 1.68	NS	24.39	25.11	− 0.87	NS
Expert	11.49	16.77	− 1.96	0.05	11.37	11.58	− 1.88	NS

Medians and Wilcoxon signed-rank test of the novices, intermediates and experts

**Table 3:** Comparing the seventh and ninth laparoscopic and robotic suturing trials

	RAS—LS				LS—RAS			
	Trial 7	Trial 9	*Z* value	*p* value	Trial 7	Trial 9	*Z* value	*p* value
Time (s)								
Novice	274.10	231.28	− 1.244	NS	182.92	141.88	− 1.245	NS
Intermediate	90.82	114.86	− 1.580	NS	100.21	126.0	− 0.051	NS
Expert	80.15	64.60	− 1.350	NS	84.52	49.83	− 2.201	0.028
Max. force (N)								
Novice	4.09	4.13	− 1.481	NS	4.84	4.63	− 0.356	NS
Intermediate	4.39	4.25	− 0.357	NS	5.07	9.09	− 2.091	0.037
Expert	4.39	4.58	− 0.471	NS	3.57	3.37	− 1.153	NS
Mean NZ force (N)								
Novice	1.07	0.84	− 0.474	NS	1.46	1.05	− 0.890	NS
Intermediate	1.14	1.32	− 0.051	NS	1.20	1.88	− 0.764	NS
Expert	1.05	1.06	− 0.345	NS	0.95	1.11	− 1.261	NS
Max. impulse (N/s)								
Novice	35.63	21.88	− 0.533	NS	28.38	18.38	− 0.889	NS
Intermediate	26.75	32.50	− 0.255	NS	25.11	41.87	− 0.764	NS
Expert	16.77	14.70	− 1.161	NS	11.58	16.44	− 1.153	NS
Force volume (N^3^)								
Novice	0.98	0.62	− 0.178	NS	3.25	1.25	− 0.044	NS
Intermediate	0.92	0.96	− 0.866	NS	1.16	5.98	− 0.357	NS
Expert	0.83	0.68	− 0.722	NS	0.74	0.97	− 0.943	NS

Medians and Wilcoxon signed-rank test of the novices, intermediates and experts

## Discussion

This study shows the changes in technical skill and tissue handling behaviour when switching between RAS and LS, according to objective force-based parameters. An increase in applied forces, representing decay in tissue handling skills, is observed in the less experienced groups when switching from LS to RAS. Where an increase in maximum force-parameters (Figs. 3, 6) indicates force-interaction errors, the increase in mean non zero force-parameter indicates a structural high load of the tissue during tasks that require constant traction [19, 22]. When switching from the RAS to LS, the max impulse outcomes increases (Fig. 5), indicating that the energy transmitted from the instrument to the tissue increases drastically [22]. If these parameter increases during suturing, cutting or dissecting, the risk on tissue damage or rupture also increases [31]. Moreover, excessive force application to the tissues can cause serious complications, even leading to bowel perforation and sepsis [32, 33]. An ex vivo experimental study using force measurements showed that the average acceptable force varied between 1.25 and 11.43 N, depended on the type of tissue [31]. Also, grasping forces can cause unwanted serosal thickness and histopathological changes that lead to think about force-based safety thresholds [32, 34]. These results are in line with another crossover study by Omar Hassan et al. [33], were novices had similar learning curves for RAS and LS, with limited crossover effect between approaches. Besides, like our results, this study also reported increased excessive forces in the RAS trials, compared to LS trials.

The influence of laparoscopic experience on the robotic performance has been marginally reported in previous studies. In 2018, Pimentel et al. [35], conducted a study comparing novices and experts performing tasks on a virtual reality simulator. There was no significant difference in any of the objective metrics between the differently experienced groups and there was no evidence regarding the transferability of laparoscopic skills to robotic-assisted surgery. Similarly, in 2012, Kilic et al. [36, 37] compared two groups with different experience in laparoscopy. The groups performed a knot tying task in a LS and RAS setting and the total completion time was the only objective metric. The more experienced trainees had a significant difference in the total completion time during the RAS trials. However, despite the difference in prior laparoscopic experience, this difference was not visible in the LS trials.

The present results show that intermediates and novices perform better at the end of their learning curve when starting with RAS, compared to groups that started with LS. These changes in technical skills are likely related to the difference in controls of both systems and should be considered when training skills. From human–machine interaction studies, it is observed that a change in interface requires the operator to switch from a more automated modus, to a more complex neuro control part of the brain that require more active thinking, which may cause problems with execution of the tasks [36–38]. The novices and experts experienced less decay of tissue handling skills (MaxForce) when switching from LS to RS, compared to intermediates. The variation in task time in trial seven to nine (Table 3), compared to trial four to six (Fig. 2), indicates that more novices and intermediates had difficulties with efficient suturing performance after changing from RAS to LS. However, when switching from LS to RAS, novices and intermediate outcomes are comparable between techniques, but expert data shows that task time increased for many surgeons. These outcomes can be explained by a lack of haptic feedback in RAS, compared to conventional LS.

Likewise LS, the adaptation of RAS and the implementation of it in hospitals is usually initiated by experienced surgeons. We know that even expert surgeons can apply significantly higher force during LS, compared to open surgery [39]. This is caused by the loss of tactile and haptic feedback. Although RAS has the advantage in accuracy and more precise handling of instruments, the sensory feedback mechanism are further diminished [6–8], which results in dangerously high force application on the tissues. Haptic force feedback during training has the potential to limit the applied intracorporeal forces, with a decrease up to 44% [8]. This could also potentially be explained by the fact that novices have not developed the neural network and spatiotemporal abilities, such as dealing with long instruments and the fulcrum effect, needed for safe minimally invasive surgery. Intermediates (senior residents and young surgeons) are potentially disadvantaged by the acquired laparoscopic skills during residency, while transitioning to RAS. Similar to our results, a recent study on robotic bowel anastomosis, showed lower anastomosis quality and lower Global Evaluative Assessment of Robotic Skills (GEARS) scores after laparoscopic experience, compared to only open surgery experience [40].

Shortly after the introduction of simulation training for LS, assessment forms like Objective Structured Assessment of Technical Skills (OSATS) haven been used to determine technical competency. These forms have been a reliable tool for the qualitative assessment of a procedural performance for over two decades, but are money and time consuming, as the trainees are usually assessed by senior surgeons. With rapid technological innovations, this type of procedural assessment was then followed by systems that recorded motion analysis parameters. Unfortunately, these forms and measures of time and motion efficiency (i.e., instrument handling), provide limited quantitative information on the effect that these instruments have on the tissue. GEARS might be suitable to detect improvement of basic skills over time in inexperience trainees [41, 42]. However, there is no association between laparoscopic experience and robotic-assisted suturing performance, according to OSATS scores, and there is evidence that assessment forms are not be suitable for the assessment of robotic technical skills and to differentiate between levels of expertise [43, 44]. This was confirmed by other recent studies, were novices were assessed during RAS skills training [45, 46]. The GEARS increased self-awareness, but did not influence the acquisition of technical skill among trainees, and no correlation was found between technical skill and operative performance. This shows that besides technical skills, also cognitive skills, intraoperative strategies and decision making should be trained before commencing RAS in the OR.

In contrast, during a study on robotic inguinal hernia repair, objective robot generated performance metrics have been shown to be accurate and more reliable than self-awareness or even faculty surgeons assessment [47]. This was also concluded by our previous work regarding the use of objective force-based parameters to assess technical skills during laparoscopic skills training [29, 30, 48]. Following force-based assessment tools for LS, validity evidence for objective assessment tools for RAS in accumulating [15, 16, 49–52]. A recent construct validation study by our group confirmed the potential for force-based parameters in assessment of tissue handling skills in RAS training [51]. Those results, along with the present results, underline the importance of quantitative objective force-based assessment of tissue-tool interaction and tissue manipulation skills. These parameters can now be utilized not only for personalized assessment and feedback during training, but also serve as benchmarks for group training and for proficiency-based training.

## Strengths and limitations

In this multicenter study, conducted at renowned academic training centers for training in minimally invasive surgery, a large cohort with 60 participants of three different experience levels performed a standardized surgical knot tying task improving the scientific value of the results. Technical skill was assessed by the recently for RAS validated ForceSense system that, in constrast to time parameter and GEARS, can objectively assess the forces applied to the tissue [51]. Using a standardized suturing and knot tying as task adds validity to the experiments as this task is representative for the assessment and validation of laparoscopic and general surgical skill [53, 54]. Furthermore, the use of a real DaVinci system, real instruments with haptic feedback, and a realistic suturing task, simulated a setting which increased the generalizability and suggest the transferability of these outcomes to the operating room. Although it added value to the generalisability of the results, this study is limited by variability and the heterogeneity of the subgroups. If inclusion criteria were more strict, these data could also be used to identify learning curve effects between groups. As is clear from the results and from the literature review, during the interpretation of these data, one should consider in which context the data have been acquired. For example, the level of experience, the complexity of the tasks and de construct validity of the assessment tool that is used. Furthermore, no motion parameters that represent instrument handling skills were included in this study. As the DVR system of Davinci and ForceSense system use different assessment parameters. Therefore following studies should solve this issue or allow the use of robotic instruments inside the ForceSense system, by increasing its diameter.

## Conclusion

The crossover effects in technical skills between laparoscopic and robot-assisted surgery are highly depended on the prior experience with laparoscopic surgery. Experts can alternate between approaches without impairment of technical skills. Less experienced surgeons showed decreased efficiency of motion when switching from robotic to laparoscopic surgery, and high tissue handling forces when switching from laparoscopic to robotic surgery. Additional simulation training is advised for these trainees to prevent from undesired events.

## Supplementary Information

Below is the link to the electronic supplementary material.

Supplementary file1 (DOCX 30 KB)
