# Supplementary material for: Crossover-effects in technical skills between laparoscopy and robot-assisted surgery
Source: Surg Endosc. 2023 Apr 25;37(8):6015–24. doi: 10.1007/s00464-023-10045-6 (PMC10338573; doi:10.1007/s00464-023-10045-6)
Supplement: Supplementary file 1 — Supplementary file1 (DOCX 30 KB) [file 464_2023_10045_MOESM1_ESM.docx]

***Supplemental files***

***Table A RAS – LS group laparoscopic and robotic suturing trials***

*Medians and and Wilcoxon signed-rank test of the novices, intermediates and experts.*

|  | **Robot** | | | |  | **Laparoscopy** | | | |
| --- | --- | --- | --- | --- | --- | --- | --- | --- | --- |
|  | Trial 1 | Trial 6 | Z value | p-value |  | Trial 7 | Trial 12 | Z value | p-value |
| Time (s) |  |  |  |  |  |  |  |  |  |
| Novice | 155,38 | 86,72 | 1,54 | NS |  | 274,10 | 181,37 | -1,82 | NS |
| Intermediate | 109,45 | 87,31 | -1,58 | NS |  | 90,82 | 79,84 | -2,395 | **0,01** |
| Expert | 110,10 | 75,59 | -2,67 | **0,01** |  | 80,15 | 60,86 | -2,83 | **0,01** |
| Max. force (N) |  |  |  |  |  |  |  |  |  |
| Novice | 7,36 | 4,46 | -2,52 | **0,01** |  | 4,09 | 5,19 | -0,42 | NS |
| Intermediate | 6,08 | 5,38 | -0,76 | NS |  | 4,39 | 4,58 | -0,459 | NS |
| Expert | 5,09 | 4,42 | -1,10 | NS |  | 4,39 | 3,14 | -2,062 | **0,04** |
| Mean NZ force (N) |  |  |  |  |  |  |  |  |  |
| Novice | 1,29 | 1,17 | -1,82 | NS |  | 1,07 | 1,18 | -0,84 | NS |
| Intermediate | 1,20 | 1,22 | -0,26 | NS |  | 1,14 | 1,20 | -0,051 | NS |
| Expert | 1,15 | 1,05 | -1,88 | NS |  | 1,05 | 0,98 | -2,413 | **0,02** |
| Max. impulse (N/s) |  |  |  |  |  |  |  |  |  |
| Novice | 25,63 | 14,43 | -2,52 | **0,01** |  | 35,63 | 21,23 | -0,7 | NS |
| Intermediate | 18,35 | 16,32 | -1,78 | NS |  | 26,75 | 27,16 | -1,172 | NS |
| Expert | 16,06 | 11,49 | -1,96 | **0,05** |  | 16,77 | 9,81 | -2,271 | **0,02** |
| Force volume (N^3^) |  |  |  |  |  |  |  |  |  |
| Novice | 1,96 | 1,50 | -2,52 | **0,01** |  | 0,98 | 0,96 | -0,56 | NS |
| Intermediate | 1,67 | 1,25 | -0,56 | NS |  | 0,92 | 0,68 | -0,255 | NS |
| Expert | 1,21 | 1,34 | -1,73 | NS |  | 0,83 | 0,65 | -1,992 | **0,05** |

***Table B LS – RAS group laparoscopic and robotic suturing trials***

*Medians and and Wilcoxon signed-rank test of the novices, intermediates and experts.*

|  | **Laparoscopy** | | | |  | **Robot** | | | |
| --- | --- | --- | --- | --- | --- | --- | --- | --- | --- |
|  | Trial 1 | Trial 6 | Z value | p-value |  | Trial 7 | Trial 12 | Z value | p-value |
| Time (s) |  |  |  |  |  |  |  |  |  |
| Novice | 205,02 | 134,28 | -2,76 | **0,01** |  | 182,92 | 112,58 | -1,33 | NS |
| Intermediate | 100,78 | 68,68 | -0,76 | NS |  | 100,21 | 99,40 | -1,99 | **0,04** |
| Expert | 81,76 | 44,17 | -2,20 | **0,03** |  | 84,52 | 34,21 | -1,83 | NS |
| Max. force (N) |  |  |  |  |  |  |  |  |  |
| Novice | 5,79 | 5,40 | -0,45 | NS |  | 4,84 | 5,73 | -0,36 | NS |
| Intermediate | 6,02 | 6,21 | -0,76 | NS |  | 5,07 | 5,83 | -0,15 | NS |
| Expert | 4,06 | 3,90 | -0,52 | NS |  | 3,57 | 3,35 | -0,37 | NS |
| Mean NZ force (N) |  |  |  |  |  |  |  |  |  |
| Novice | 1,08 | 1,14 | -0,49 | NS |  | 1,46 | 1,22 | -1,16 | NS |
| Intermediate | 1,32 | 1,45 | -0,71 | NS |  | 1,20 | 1,62 | -0,42 | NS |
| Expert | 0,97 | 1,15 | 0 | NS |  | 0,95 | 0,85 | -1,46 | NS |
| Max. impulse (N/s) |  |  |  |  |  |  |  |  |  |
| Novice | 35,29 | 21,85 | -0,45 | NS |  | 28,38 | 20,45 | -1,07 | NS |
| Intermediate | 19,86 | 24,39 | -0,46 | NS |  | 25,11 | 17,78 | -0,05 | NS |
| Expert | 22,05 | 11,37 | -0,94 | NS |  | 11,58 | 7,83 | -1,46 | NS |
| Force volume (N^3^) |  |  |  |  |  |  |  |  |  |
| Novice | 1,10 | 1,79 | -0,62 | NS |  | 3,25 | 1,88 | -0,53 | NS |
| Intermediate | 1,08 | 2,51 | -1,07 | NS |  | 1,16 | 2,07 | -0,15 | NS |
| Expert | 0,74 | 0,76 | -0,94 | NS |  | 0,76 | 0,47 | -2,02 | **0,04** |
